# PAIN INTENSITY AND INTERFERENCE PREDICT STAGE OF ENGAGEMENT IN SELF-MANAGEMENT IN OLDER AFRICAN AMERICAN ADULTS

**DOI:** 10.1093/geroni/igad104.2803

**Published:** 2023-12-21

**Authors:** Staja Booker, Ann Horgas, Jianli Wu

**Affiliations:** University of Florida, Gainesville, Florida, United States; University of Florida, Gainesville, Florida, United States; University of Florida, Gainesville, Florida, United States

## Abstract

Osteoarthritis in African American older adults often causes a disproportionate burden of severe chronic joint pain that interferes with physical and emotional/cognitive activities. One tool commonly used in clinical research studies to evaluate patterns in pain intensity and pain interference is the Brief Pain Inventory-SF (BPI-SF). Confirmatory factor analysis validated a three-factor structure of the BPI-SF comprising pain intensity, activity-related pain interference, and affective pain interference. However, what is unclear and novel, is whether these three factors predict stage of readiness to engage in osteoarthritis self-management. Stage of readiness to engage (pre-contemplation, preparation, action) were assessed for seven recommended osteoarthritis self-management strategies: land-based exercise, water exercise, strength training/stretching, assistive devices, warm/cool compresses, medications, and self-management education. Using secondary data from 110 African American men and women (Mean Age 68.4, SD= 12.4), we performed ANOVA and ordinal logistic regression in SPSS. Pain intensity, activity-related pain interference, and affective pain interference were not significantly different between stages of engagement in pain self-management strategies except for medication use and assistive devices (p<.05). After controlling for age and employment, higher pain intensity was significantly associated with 27% reduction in the odds of being in preparation phase for water exercise, and higher levels of activity interference was associated with a 42% increase in the odds of being in the action phase for assistive devices. These results help us understand the relationship between behaviors and pain, particularly the impact of pain intensity and interference with physical activities on which strategies African Americans select to manage pain.

